# Diagnostic Value of Hysterosalpingography and Laparoscopy for Tubal Patency in Infertile Women

**DOI:** 10.5812/nms.10661

**Published:** 2013-06-27

**Authors:** Fatemeh Foroozanfard, Zohreh Sadat

**Affiliations:** 1 Department of Gynecology and Obstetrics, Kashan University of Medical Sciences, Kashan, IR Iran; 2 Trauma Nursing Research Center, Kashan University of Medical Sciences, Kashan, IR Iran

**Keywords:** Fallopian tube patency, Diagnosis, Infertility, Hysterosalpingography, Laparoscopy

## Abstract

**Background:**

Tubal occlusion is one of the most frequent causes of infertility in women. The evaluation of the fallopian tube is necessary to determine the management plan for infertility. The two most important diagnostic procedures which are used for the evaluation of tubal patency are hysterosalpingography (HSG) and laparoscopy.

**Objectives:**

The aim of this study was to compare HSG and laparoscopic findings in the diagnosis of tubal patency.

**Patients and Methods:**

In a prospective study sixty two infertile cases were examined by HSG as part of their routine infertility evaluation, three months after HSG, tubs status were assessed by laparoscopy as a gold standard method. The findings of HSG and laparoscopy were compared. The Laparoscopy findings were used as reference standard to calculate sensitivity, specificity, positive and negative predictive values for unilateral and bilateral no tubal patency.

**Results:**

The sensitivity and specificity of HSG on bilateral tubal patency or no bilateral tubal patency were 92.1% and 85.7% respectively. The positive and negative predictive values were 97.2% and 66.7%, and the accuracy was 91.1%. The sensitivity and specificity of HSG for evaluation of the bilateral tubal patency and unilateral or bilateral no tubal patency were 77.8% and 52.94%, the positive and negative predictive values were 81.4% and 47.4% respectively, and the accuracy was 71%.

**Conclusion:**

HSG is considered to have a high sensitivity and specificity. HSG and laparoscopy are not alternative, but are the complementary methods in the examination of no tubal patency.

## 1. Background

Approximately 5% of the couples are definitive infertile with a nearly zero chance of becoming spontaneously pregnant in the future (1). Fertility varies across regions of the world and is estimated to affect 8 to 12 percent of couples worldwide (2). Total infertility is divided into primary and secondary infertility. Primary infertility is defined as the inability to conceive within one year of exposure to pregnancy among women 15 to 49 years old with sexually active and non-contraception. Secondary infertility refers to the inability to conceive following a previous pregnancy (3). For many couples, infertility and its treatment cause a serious strain on their interpersonal relationship, and cause disturbed relationships with other people (4). Tube blockage is one of the most frequent causes of infertility in women. One third of infertility cases are due to anatomical abnormalities of the female reproductive tract such as tubal blockage (5, 6). The degree of tubal pathology determines the possibility for fertility. The evaluation of the fallopian tube is necessary to determine the management plan of infertility. A number of diagnostic tests are being used in clinical practice to assess tubal patency as part of the work-up for subfertility (7).

The most commonly used tests are hysterosalpingography (HSG) and laparoscopy. The HSG is a contrast enhanced fluoroscopic and flat plate study used to evaluate the endometrial cavity and fallopian tubes. It has been a test in the workup of infertile couples as a minimally invasive method of evaluating tubal patency and is performed as the first line approach for assessing tubal pathology. Whereas laparoscopy is considered the clinical reference test for diagnosing tubal pathology (8). Laparoscopy allows visualization of peri-adnexal adhesions and the presence of endometriosis, which cannot be done with HSG (9).

In a research, HSG was compared with laparoscopy, and the results showed that sensitivity was 65% for tubal patency but it increases the achievement of spontaneous pregnancy by three times (10). Another study showed HSG may be used as a screening test for tubal patency. Results demonstrates high specificity of HSG for diagnosis of tubal occlusion and low sensitivity in cases with peri-tubal adhesions (11). Results by Sakar et al. showed the sensitivity and specificity of HSG were 63% and 89.3% respectively, and the positive predictive value was 92%, with a 55% negative predictive value, and the accuracy ratio was 72% (9), in this study researchers concluded that these two methods are complementary. In another study, laparoscopy was found to be a better predictor of future fertility than HSG. Also in women who are at an increased risk of having tuboperitoneal pathology, diagnostic laparoscopy should be offered early in the infertility work-up (12). There are conflicting results about the diagnostic value of HSG. 

## 2. Objecives

The aim of this study was to compare HSG and laparoscopic findings in the diagnosis of tubal patency.

## 3. Patients and Methods

In a prospective study, the cases that were followed for infertility in Shahid Beheshti Hospital in Kashan, Iran, were evaluated between 2007 and 2008. Inclusion criteria were no prior pelvic surgery, no history of pelvic infection, normal bimanual pelvic examination, normal semen parameters of partner, no ovulatory dysfunction, and excluding criteria were surgical procedures that had occurred between the performance after HSG, women who did not return for laparoscopy evaluation, technical problems related to HSG and women who became pregnant after hysterosalpingography. During the study, sixty two patients seen at our center underwent HSG as part of the evaluation or treatment of infertility and all were included in this study. Laparoscopy was performed three month after HSG (13). The HSG was performed by radiologist. The procedure was performed between days 6 and 12 of the menstrual cycle at least 48 hours after menses had ceased. The women were advised to avoid unprotected intercourse in this period. HSG was performed using a sterile technique. The patients were placed in a lithotomy position, and a vaginal speculum was inserted. The balloon catheter was inflated within the endocervical canal or lower uterine cavity. Approximately 10–15 mL of a water-soluble contrast were injected manually through the cannula. Fluoroscopic examination was performed during the injection. Three x- ray films were taken; images of early and maximal opacification of the uterine cavity, fallopian tubes, and peritoneal contrast spillage were obtained. Prophylactic antibiotics were prescribed. The patients were routinely pre-medicated prior to the procedure with oral mefenamic acid 500 mg three times per day until 48 hours after the procedure. The results of HSG were evaluated by radiologists.

Laparoscopy was performed under a general anaesthesia by an infertility specialist (the first researcher). A one cm incision was made within or just below the lower edge of the umbilicus. Through this incision the abdominal cavity is inflated with carbon dioxide gas and pneumoperitoneum being obtained. A trocar was inserted in the same region. The cannula of the trocar was left, and the trocar was pulled out. Then a laparoscope was introduced through the cannula. The abdominal cavity and pelvic were examined in the trendelenburg position. A traumatic grasper forceps were used by the assistance of a second trocar for better visualization. A third trocar was applied if required. To assess tubal patency, methylene blue was injected through another uterine manipulator and results of laparoscopy were recorded by the infertility specialist as well. Demographic characteristics were collected through the interview using a structured questionnaire. Results of HSG and laparoscopy were also recorded in a check list by a trained midwife. Variables were age, primary and secondary infertility, duration of infertility, job, education, tubal patency (yes or no), bilateral no tubal patency and unilateral no tubal patency. Primary infertility describes couples who have never been able to become pregnant after at least one year of unprotected sexual cohabitation. Secondary infertility describes couples who have been pregnant at least once, but have not been able to become pregnant again. The Laparoscopy findings were used as a reference standard to calculate sensitivity, specificity, positive and negative predictive values for bilateral tubal no patency and unilateral or bilateral tubal no patency. Data was analyzed by SPSS software (version 16).

The study protocol was approved by the local research ethics committee at Kashan University of Medical Sciences. Patients were enrolled after having provided their informed written consent. Participants were assured that their information would be kept confidential and provisions of the Helsinki Convention were considered.

## 4. Results

Sixty-two patients were included in the study consisted of 43 (69.3%) cases of primary infertility and 19 (30.7%) cases of secondary infertility. Mean duration of primary and secondary infertility were 5.79 ± 3.19 and 5.97 ± 3.36 years respectively. Maximum number of cases had duration of infertility between 1 to 4 years (45.2 %). The average age in subjects of primary infertility were 26.25 ± 3.85 years and in subjects of secondary infertility were 29.73 ± 4.87 years. Up to 70% of cases had a high school or less than high school education and 92% of women were not employed.

Forty three cases had normal HSG, among them 81.4% had normal laparoscopy. In the nineteen cases with abnormal HSG (unilateral or bilateral no patency), 47.4 % of patients showed abnormal results on laparoscopy. HSG showed a bilateral tubal patency in 69.4 % and unilateral or bilateral no patency in 30.6 %. Laparoscopy showed a bilateral tubal patency in 72.6 % and unilateral or bilateral no patency in 27.4 %. (Table 1).HSG showed bilateral patency in 36 cases, while laparoscopy revealed bilateral patency in 35 cases. The HSG and laparoscopy revealed bilateral no patency in nine and three cases respectively. The sensitivity of HSG on bilateral tubal patency or no bilateral tubal patency was 92.1% and its specificity was 85.7%. The positive predictive value and the negative predictive value were 97.2 % and 66.6% respectively. Furthermore, results of HSG were false-negative in 33.3% of patients, false-positive in 2.8% (Table 2) and accuracy was 91.1%. The sensitivity and specificity of HSG on bilateral tubal patency and any abnormality of patency (unilateral or bilateral tubal no patency) were 77.8% and 52.9% respectively, the positive predictive value and the negative predictive value were 81.4 % and 47.4% respectively. Furthermore, results of HSG were false-negative in 52.6% of patients, false-positive in 18.6% (Table 3) and accuracy was 71%.

**Table 1.** Tubal Status (Bilateral Patency, Unilateral and Bilateral No Patency) Detected by Hysterosalpingography and Laparoscopy

Hysterosalpingography	Laparoscopy			Total
	Bilateral tubal patency, No. (%)	Unilateral tubal patency, No. (%)	Bilateral tubal no patency, No. (%)	
**Bilateral tubal patency**	35 (56.5)	7 (11.3)	1 (1.6)	43 (69.4)
**Unilateral tubal patency**	7 (11.3)	2 (3.2)	-	9 (14.5)
**Bilateral tubal no patency**	3 (4.8)	1 (1.6)	6 (9.7)	10 (16.1)
**Total**	45 (72.6)	10 (16.1)	7 (11.3)	62 (100)

**Table 2.** Diagnostic Value of Hysterosalpingography and Gold Standard Laparoscopy in Bilateral Tubal Patency and Bilateral Tubal No Patency

Hysterosalpingography	Laparoscopy			
	Bilateral tubal patency, No.(%)	Bilateral tubal no patency, No.(%)	Total	Diagnostic Value, %
**Bilateral tubal patency**	35 (97.2)	1 (2.8)	36 (100)	Positive predictive value 97.2
**Bilateral tubal no patency**	3 (33.3)	6 (66.7)	9 (100)	Negative predictive value 66.6
**Total**	38 (84.4)	7 (15.6)	45 (100)	
**Diagnostic value, (%)**	Sensitivity 92.1	Specificity 85.7		

**Table 3.** Diagnostic Values of Hysterosalpingography and Gold Standard Laparoscopy in Bilateral Tubal Patency, Unilateral or Bilateral Tubal No Patency

Hysterosalpingography	Laparoscopy			
	Bilateral tubal patency, No. (%)	Unilateral or bilateral tubal no patency, No. (%)	Total	Diagnostic value, %
**Bilateral tubal patency**	35 (81.4)	8 (18.6)	43 (100)	Positive predictive value 81.4
**Unilateral or bilateral tubal no patency**	10 (52.6)	9 (47.4)	19 (100)	Negative predictive value 47.4
**Total**	45 (72.6)	17 (27.4)	62 (100)	
**Diagnostic value, %**	Sensitivity 77.8	Specificity 52.9		

## 5. Discussion

In this study we compared findings of HSG and laparoscopy in infertile women in regard to the tubal patency. The estimated sensitivity and specificity were 92.1% and 86.7% respectively for HSG, when definition of tubal occlusion was limited to bilateral no patency. When no tubal patency was defined as any abnormality of tubal patency (unilateral or bilateral no patency), sensitivity and specificity were 77.8% and 52.9% respectively, the results showed HSG is more accurate in the diagnosis of tubal bilateral no patency. These findings are comparable to a number of studies. In a study by Tvarijonaviciene et al, sensitivity of 84.1% and specificity of 59.1%, were calculated when tubal occlusion was defined as any abnormality of tubal patency. When definition of tubal occlusion was limited to two-sided occlusion, the sensitivity and specificity were 89.5% and 90% respectively (13). Diagnostic value of HSG and laparoscopy in hundred and two infertile women was evaluated by Vasiljevic et al. and the concordant findings by HSG and laparoscopy in unilateral tubal blockage were found in 61.5% of cases, and in bilateral tubal blockage in 70.4% of women. The total concordant findings by HSG and laparoscopy in tubal blockage were found in 65.7 of cases (14); results of the two above studies are consistent with our findings. In a Meta analysis, accuracy of HSG was evaluated, using findings of laparoscopy as the reference. Sensitivity and specificity of HSG were 53% and 87% for any tubal pathology and 46% and 95% for bilateral tubal pathology. (15) Also in another retrospective study, medical records of women admitted to a local Iranian hospital were evaluated. The findings were compared in regard to tubal obstruction. Results showed that the accuracy of HSG reports in reference to laparoscopy was 75%, sensitivity and specificity of HSG were 0.92 and 0.70, respectively (8). In this study, results reported as normal or abnormal findings and bilateral or unilateral no patency were not considered.

In a cohort study, eighty two infertile cases were evaluated to compare tuboperitoneal factors by HSG and laparoscopy. Results showed that pathological findings were observed in 45.1% by HSG and 65.85% by laparoscopy. The sensitivity and specificity of HSG were 63% and 89.3%, and the positive predictive value was 92%, with a 55% negative predictive value, and the accuracy ratio was 72% (9). In other research, sixty eight patients in the initial stage of the infertility treatment were examined by further status of the tubes by Hysterosonosalpingography and gold standards laparoscopy. For the assessment of tubal patency using positive contrast, the method has shown 100% sensibility and 77% specificity, the negative predictive value and positive predictive value were 100% and 70% (16). In this study, Hysterosonosalpingography as a contrast ultrasound method was performed that might show different results from HSG. A research was performed by Shah and et al (17) to evaluate and compare the diagnostic value of HSG and laparoscopy; fifty patients were initially diagnosed with either unilateral or bilateral tubal blockage using HSG. Six to eight weeks later, tubal patency was assessed by laparoscopy. HSG diagnosed bilateral proximal, bilateral distal and mixed tubal occlusion in 40.5%, 35.1% and 13.5% cases respectively. In this study, HSG demonstrated a reduced positive predictive value especially for bilateral proximal tubal occlusion. In this study, patients were initially diagnosed with either unilateral or bilateral no tubal patency using HSG later evaluated by laparoscopy, while in our study infertile women who were diagnosed with tubal patency or no patency were included. 

Also in another study, HSG and laparoscopic findings regarding normal tubes were in agreement in 22.9%. The sensitivity of HSG was observed in detection of proximal tubal occlusion (78%). The specificity of HSG was observed in the diagnosis of combined occlusions (96%) (18). 

The possible reasons for the difference in results might be due to tubal spasm and endometrial polyp in the area of the uterine opening of the tubes, also anatomic variations in the tubes.

HSG is a simple method for examination of female sterility, a more economical and elementary method suitable for evaluation of tubal pathologies and it seems to be in identification of some congenital uterine anomalies. The advantage of laparoscopy is identified by the possibility of visualization of some other pelvic abnormalities which may be the cause of infertility and cannot be detected by HSG such as endometriosis, adhesions, tuberculosis. However, limitation of this study was that the interpretation of the images of HSG depends on the experience and ability of the radiologists involved. 

In conclusion, these two methods are not alternative, but are the complementary methods in the diagnosis of tubal patency.
